# Improvement of Osseointegration by Ultraviolet and/or Simvastatin Treatment on Titanium Implants with or without Bone Graft Materials

**DOI:** 10.3390/ma14133707

**Published:** 2021-07-02

**Authors:** Ji Hoon Jun, Kyung Chul Oh, Kyu-Hyung Park, Narae Jung, Jiayi Li, Hong Seok Moon

**Affiliations:** 1 Department of Prosthodontics, Yonsei University College of Dentistry, Seoul 03722, Korea; jhjun0103@yonsei.ac.kr (J.H.J.); kyungabc@yuhs.ac (K.C.O.); ljyzoeli24@gmail.com (J.L.); 2 Aeromedical Squadron, Republic of Korea Air Force 8th Fighter Wing, Wonju 26304, Korea; 3 Oral Science Research Center, BK21 Plus Project, Yonsei University College of Dentistry, Seoul 03722, Korea; khyungpark@gmail.com (K.-H.P.); jnrgood1217@yuhs.ac (N.J.)

**Keywords:** animal experiments, bone implant interactions, bone substitutes, bone regeneration, statin, illumination, titanium

## Abstract

We evaluated and compared ultraviolet (UV) treatment and simvastatin (SIM) immersion effects on the osseointegration of sandblasted, large-grit, acid-etched (SLA) titanium dental implants at two different time points in rabbit tibias, with or without xenogenic bone graft materials. The surface alteration on simvastatin treatment titanium discs was analyzed using an infrared spectrometer. Implants were categorized into four groups according to the surface treatment type. Twelve rabbits received two implants per tibia. A tibial defect model was created using a trephine bur, with implants in contact with the bone surface and bovine bone graft materials for gap filling. The rabbits were sacrificed after 2 or 4 weeks. UV treatment or SIM immersion increased the bone-to-implant contact (BIC) on nongrafted sides, and both increased the BIC and bone area (BA) on grafted sides. The application of both treatments did not result in higher BIC or BA than a single treatment. At two different time points, BIC in the nongrafted sides did not differ significantly among the UV and/or SIM treated groups, whereas BA differed significantly. UV or SIM treatment of SLA titanium implants accelerates osseointegration in tibias with or without xenogenic bone graft materials. The combination of both treatments did not show synergy.

## 1. Introduction

Immediate implant placement is the insertion of a dental implant directly into a fresh extraction socket site [1]. Its advantages include fewer surgeries, shorter total treatment time, less crestal bone loss, and favorable esthetic outcomes [2]. Implants are usually placed in contact with the palatal bony wall to keep the labial wall intact and achieve favorable initial stability in the maxillary anterior region [3]. Consequently, in most cases, a gap defect is formed around the coronal area between the labial wall and the implant due to the discrepancy in size and shape between the implant and the socket [4]. Inserting xenogenic bone graft materials such as bovine bone that provide structural support for new bone formation in conjunction with a resorbable membrane into these gaps has been proven to be an effective and predictable approach [1,5,6,7,8].

Osseointegration is defined as direct contact at the microscopic level between living bone tissue and an implant without interposed soft tissue [9,10,11,12,13]. Attaining favorable implant stability is a major factor in successful osseointegration. Primary stability is obtained by mechanical engagement of the implant threads with bone tissue during implant installation and is influenced by bone quality and quantity, surgical technique, and implant thread design [14]. By contrast, secondary stability is derived from bone regeneration during healing, which dictates the time of functional loading, and is closely related to bone-to-implant contact (BIC) [15,16].

BIC is predominantly influenced by implant surface characteristics. With advances in implant dentistry, a number of methods for increasing BIC have been reported. Among the most popular methods of modifying an implant surface is the roughening of the surface using the sandblasted, large-grit, acid-etched (SLA) technique [17]. The SLA surface adequately differentiates preosteoblasts, reinforces the osseointegration process, and results in a higher BIC than achieved with a smooth, turned surface [18,19]. Another adjunctive method to increase BIC is a physicochemical modification of implant surfaces by the use of ultraviolet (UV) irradiation, in a process termed photofunctionalization. Many studies have demonstrated that this is a simple and effective method to enhance osseointegration [20,21,22,23]. UV pretreatment of titanium implants stimulates osseous healing by rendering the surface superhydrophilic and removing hydrocarbons adsorbed on the surface [20,21,23,24,25,26,27,28,29]. Furthermore, some studies have reported improved biological capabilities of UV treatment at the cellular and genetic levels [20,29,30,31].

The application of biomaterials that can accelerate the bone healing process after implant placement has also drawn significant attention. One such example is statins [32]. Statins are cholesterol-lowering drugs that were originally developed to treat patients with cardiovascular disease. Interestingly, evidence has emerged showing that statins have beneficial effects on bone healing and turnover [33,34]. Statins act as dual agents that promote anabolic and inhibit catabolic functions in bone metabolism [35]. In vitro studies have shown that statins induce osteoblast differentiation and enhance osteoblast viability [36,37,38]. Additionally, in vivo studies have proven that statins enhance the osseointegration of titanium implants [35,39]. Possible molecular mechanisms of statins related to bone remodeling reported in the literature are various and complex [35,40,41,42,43]. Simvastatin is the most investigated statin and has been proven to be a potent agent for improving implant fixation [43,44,45]. However, most statins are degraded when administered orally due to the extensive first-pass effect in the liver, and less than 5% of the drug is available to the general circulation, with even lower amounts available to the bones [44,46]. Furthermore, statins have lower bone affinity [43]. Local statin delivery directly to the site of bone remodeling has been proposed to improve the bioavailability of statins to the bones [32,47,48].

To date, no study has compared the effects of UV treatment and simvastatin soaking or investigated the synergistic effect of the two methods in osseous healing around implants over time. Furthermore, there is no report on how the application of UV and simvastatin on implant surfaces affects osseointegration and new bone formation in peri-implant defects filled with bone graft material. Therefore, considering the necessity for reinforcing the bone repair around implants and accelerating the loading of implants, we sought to investigate how irradiating the implants with UV or immersing it in a simvastatin solution could affect bone remodeling around implants in peri-implant circumferential gap defects prepared in an animal model, mimicking immediate implant placement. The null hypothesis was that there would be no difference in BIC and new bone formation between the control and UV- and/or simvastatin-treated groups with or without bone grafting.

## 2. Materials and Methods

### 2.1. Materials

#### 2.1.1. Implant and Titanium Disc

Forty-eight SLA surface-treated internal conical-type titanium dental implants (Dentium NR line; Dentium, Suwon, Korea), measuring 3.1 mm in diameter and 7 mm in length, and two titanium discs with SLA surface, 10 mm in diameter and 2 mm in thickness, prepared from pure grade IV titanium (Dentium) were utilized in the study. A short, narrow diameter implant was selected considering the average width and thickness of the rabbit tibia, with reference to previous studies [1,27,49,50,51,52,53]. In brief, SLA surface treatments were performed by sandblasting with aluminum oxide and acid etching with hydrochloric acid. The implants were manufactured at the same time and kept in separate sealed containers.

#### 2.1.2. Bone Graft Material

Bio-Oss (Geistlich Pharma AG, Wolhusen, Switzerland) was used as the xenogenic bone graft material. Its particle size ranged from 250 to 1000 μm in diameter. This material is sterilized bovine bone with all organic components removed.

#### 2.1.3. Resorbable Membrane

GENOSS collagen membrane (Genoss, Suwon, Korea) was used as a barrier membrane. It is a sterilized and biodegradable membrane made from bovine tendon (type I collagen). The collagen component of the membrane is chemically crosslinked, delaying the collapse of the barrier and permitting sufficient time for osseous maturation with biocompatibility [54].

#### 2.1.4. Simvastatin

Solid simvastatin (Sigma-Aldrich, St. Louis, MO, USA) was reconstituted in a dimethyl sulfoxide solvent (Sigma-Aldrich) to a concentration of 25 mM, and the solution was diluted to 0.5 mM in phosphate-buffered saline (PBS; GIBCO, Thermo Fisher Scientific Inc., Waltham, MA, USA). The solution was sterilized by filtration through a 0.22 μm polyvinylidene difluoride membrane (Sartorius, Göttingen, Germany) under sterile conditions and diluted to working concentrations using PBS.

### 2.2. UV Photofunctionalization

UV photofunctionalization was performed by illuminating samples with UV light for 15 min using a specialized instrument (TheraBeam SuperOsseo, Ushio Inc., Tokyo, Japan) using an exposure time based on earlier studies [28,55]. Specifications of the device were as follows: input voltage of AC 100 to 240 V ± 10%, input current of 2.2 A in maximum, temperature of 15–30 °C, humidity of 20–70%, and altitude below 2000 m. Multiple UV lamps were utilized to create the UV light as a mixed spectrum at wavelengths of 360 nm (0.05 mW/cm^2^) and 250 nm (2 mW/cm^2^) [56]. Inside the device, the lamps were arranged to illuminate a sample in all directions homogeneously.

### 2.3. Animals

Twelve specific pathogen-free female New Zealand White rabbits (3 month old and weighing 3 ± 0.5 kg), without any genetic modifications or systemic diseases, were enrolled in this animal trial. Each rabbit was housed in an individual cage and kept under standard laboratory conditions at 24 °C ambient temperature in a 12 h dark/light cycle. The location of the cage was randomly assigned. Standard diet and water were provided ad libitum.

### 2.4. In Vivo Experimental Group Design and Sample Preparation

Forty-eight SLA implants were divided equally into control and three test groups according to the treatment methods used, as follows:

- Group C: implants placed without any treatment in rabbits sacrificed at 2 weeks (Group C-2) or 4 weeks (Group C-4);
- Group U: implants irradiated with UV immediately before implantation, but not coated with simvastatin, in rabbits sacrificed at 2 weeks (Group U-2) or 4 weeks (Group U-4);
- Group S: implants immersed in simvastatin solution for 24 h in separate sealed containers without UV exposure in rabbits sacrificed at 2 weeks (Group S-2) or 4 weeks (Group S-4);
- Group SU: implants first immersed in simvastatin solution for 24 h and then irradiated with UV immediately before surgery in rabbits sacrificed at 2 weeks (Group SU-2) or 4 weeks (Group SU-4).

**Figure 1:**
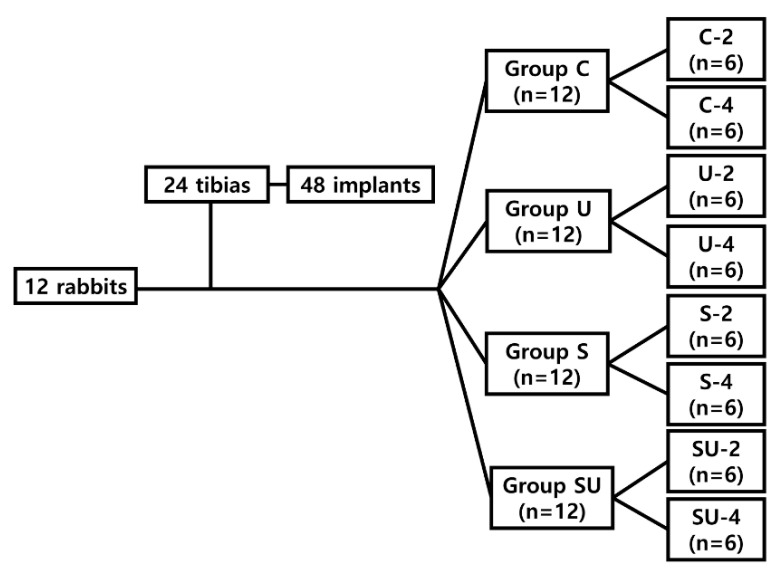
Flowchart of classification of the in vivo experimental groups. Group C, control group: implants without additional surface treatments in rabbits sacrificed at 2 weeks (C-2) or 4 weeks (C-4). Group U: implants irradiated with UV without immersion in SIM solution in rabbits sacrificed at 2 weeks (U-2) or 4 weeks (U-4). Group S: implants immersed in SIM solution for 24 h without UV exposure in rabbits sacrificed at 2 weeks (S-2) or 4 weeks (S-4). Group SU: implants first immersed in SIM solution for 24 h followed by UV irradiation before placement in rabbits sacrificed at 2 weeks (SU-2) or 4 weeks (SU-4). UV, ultraviolet; SIM, simvastatin.

**Figure 2:**
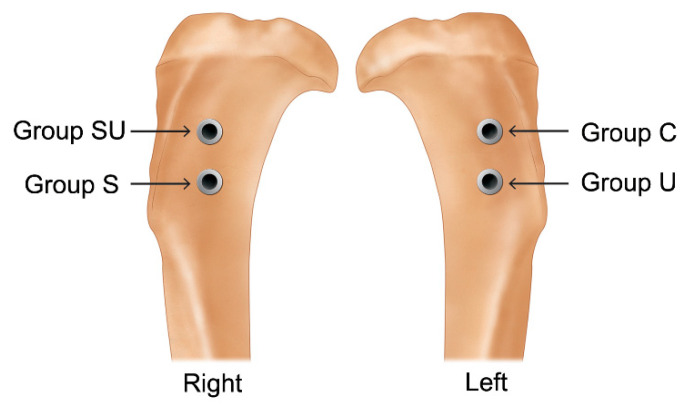
Implantation sites of four implants on the right and left proximal tibial metaphysis in a rabbit.

Each rabbit received two implants per tibia, i.e., four implants per rabbit. Group C and group U implants were placed in the left tibia, and group S and group SU implants were placed in the right tibia (Figure 1 and Figure 2). The implants were placed symmetrically to minimize differences in the surgical sites. The 12 rabbits were separated into two groups to observe differences according to the healing time. Six rabbits were allowed a healing period of 2 weeks postoperatively (2-week group) and the other six a healing period of 4 weeks (4-week group). The required sample size was calculated with reference to related studies [57,58].

### 2.5. Surface Characterization

The SLA surface-treated titanium disc and the same kind of disc, but further immersed in simvastatin solution for 24 h, were analyzed by attenuated total reflection Fourier-transform infrared (ATR-FTIR) spectrometer (Vertex 70, Bruker, Germany) to characterize the functional groups present on the surfaces. Each disc was fixed on a single reflection horizontal ATR accessory to perform analysis. FTIR spectra were measured in the wavenumber range of 4000–400 cm^−1^ at the transmittance mode, cumulating 20 scans at a resolution of 4 cm^−1^.

### 2.6. Surgical Procedure

The in vivo experiment was conducted at the Avison Biomedical Research Center. One blinded researcher (J.H.J.), unaware of the time of sacrifice and the group to which an implant belonged, placed the implants and performed bone grafts. Two blinded researchers (N.J. and J.L.) assisted the surgical procedure. One researcher (K.-H.P), aware of the group allocation of the implants, prepared implants in a separate room and offered them to the blinded researcher (J.H.J.).

After an acclimation period of 1 week, the surgical procedure was implemented as described previously [1,27,51,52,53]. General anesthesia was administered by inhalation of 2–2.5% isoflurane (Ifran, Hana Pharm Co. Ltd., Seoul, Korea), an intramuscular injection of tiletamine/zolazepam (Zoletil-50, 10 mg/kg, Virbac, Carros, France), and an intravenous injection of xylazine (2.3 mg/kg, Rompun, Bayer Korea, Seoul, Korea). The surgical site was shaved and disinfected with povidone-iodine solution. Local anesthesia was performed in the surgical area through an injection of 2% lidocaine with 1:80,000 epinephrine (2% lidocaine hydrochloride injection, Huons Co., Ltd., Seongnam, Korea).

After skin preparation and sterile draping, a 5.0 cm linear, longitudinal incision was made under aseptic conditions on the medial side of the tibia, immediately below the knee, and a full-thickness flap was raised to expose the underlying tibial bone. Two surgical circumferential defects of 5 mm in diameter and 4 mm in depth were created in the proximal surface of both the left and right tibial metaphysis, using a 5 mm diameter trephine bur (Dentium, Suwon, Korea), separated by 5 mm. Additional osteotomy was performed on the prepared bone bed using a 2.0 mm pilot drill and a 3.1 mm guide drill. A 0.9% chilled saline solution was continuously sprayed over the drill site to avoid overheating. During the osteotomy procedure, sequential drilling was performed through the opposite side of the tibia to achieve bicortical fixation of the implants. The implants were placed in contact with one side of the circular defects under 30 N·cm of torque using an electric motor with a contra-angle handpiece. The implants were engaged bicortically, and cover screws were screwed over them.

**Figure 3:**
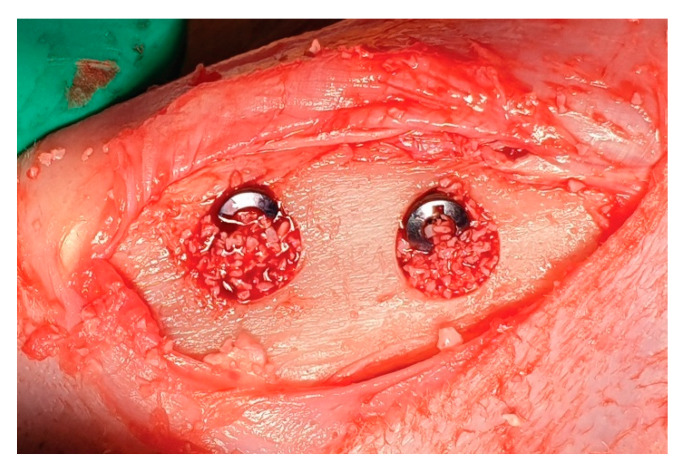
Clinical photograph illustrating implants placed in the rabbit tibia, with xenogenic graft materials placed in the gap defects.

After implant placement, bovine bone material was inserted to fill the peri-implant defects (Figure 3). A trimmed GENOSS collagen membrane was applied to cover the defect fully. The fascia and cutaneous tissue were repositioned and sutured without tension using 4-0 synthetic resorbable materials (Vicryl; Ethicon, Somerville, NJ, USA), and the skin was sutured with monofilament nylon (4-0 Monosyn, Johnson & Johnson International, Edinburgh, Scotland). A general analgesic (meloxicam, 0.1 mg/kg/day; Metacam, Boehringer Ingelheim, Ingelheim, Germany) and an antibiotic (enrofloxacin; 10 mg/kg/day, Baytril, Bayer, Seoul, Korea) were administered intravenously for 5 days postoperatively.

### 2.7. Euthanasia and Sample Collection

The rabbits were sacrificed at either 2 or 4 weeks postoperatively (6 rabbits at each time point). General anesthesia was induced by subcutaneous injection of 5 mg/kg alfaxalone (Alfaxan; Careside, Seongnam, Korea) and 0.25 mg/kg medetomidine (Tomidin; Provet Veterinary Products, Istanbul, Turkey), intramuscular injection of 2.3 mg/kg xylazine, followed by intravenous injection of 0.5 mg/kg alfaxalone and 0.12 mg/kg medetomidine. To induce euthanasia, 50 mg tramadol (Trodon injection; Ajupharm, Seoul, Korea) and 0.3 g potassium chloride (potassium chloride-40 injection; Dai Han Pharm, Seoul, Korea) were intravenously injected. The tibias were surgically reopened, and the implants and the block of bone surrounding the tibia head were harvested en bloc. The exclusion criteria for the sample were the presence of complications such as tibial fracture, infection, or inflammation.

### 2.8. Histological Processing

The samples were stored in a fixation solution for 2 weeks (10% formaldehyde solution buffered with 0.1 M phosphate solution, pH 7.2; Sigma-Aldrich). Subsequently, they were washed in running water and dehydrated in a series of increasing ethanol concentrations, in the order of 70%, 80%, 90%, and 100%. Thereafter, the dehydrated specimens, without being decalcified, were embedded in a methyl methacrylate-based resin (Technovit 7200 VLC; Kulzer & Co, Norderstedt, Germany), cured under a UV instrument (Kulzer Exact 520, Kulzer & Co, Norderstedt, Germany). Non-decalcified ground sections of the implants and surrounding bone tissue were fabricated following the method suggested by Donath and Breuner [59]. The specimens were sectioned in the longitudinal plane through the middle of the implants and reduced to a thickness of 15 μm using a microgrinding machine (Kulzer Exact 400CS, Kulzer & Co, Norderstedt, Germany). Hematoxylin and eosin staining was performed on the entirety of the slices.

### 2.9. Histomorphometric Analysis

**Figure 4:**
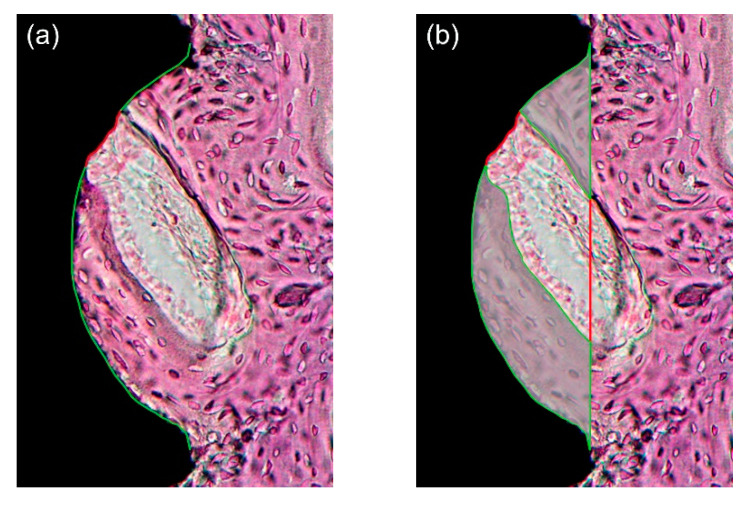
(**a**) Bone-to-implant contact (BIC, %) calculated by the length of the green lines divided by the total length of the well (green and red lines); (**b**) Bone area (BA) calculated by the area marked with green lines divided by the total area of the well. Note that the region-of-interest (ROI) was defined as an area within the three best consecutive threads engaged in the upper cortical bone, and therefore, a total of three threads were used for the calculation. Only one third of ROI is shown for simplicity.

After microscopic observation, histological images were captured at 12.5× and 40× magnification using a digital camera (Polaroid DMC2 digital microscope camera, Polaroid Corporation, Cambridge, MA, USA) attached to a light microscope (Olympus BX50; Olympus Optical, Tokyo, Japan). Quantification was performed under 40× magnification with image analysis software (ImageJ; NIH, Bethesda, MD, USA; http://imagej.nih.gov/ij/index.html, accessed on 7 February 2021). As in previous studies, an area within the three best consecutive threads engaged in the upper cortical bone region was defined as the region-of-interest (ROI) [60,61,62]. The measurement followed the method suggested by Lee et al. [29] (Figure 4). Within the ROI, the following primary outcome measures were evaluated by two blinded and trained examiners (J.H.J. and K.C.O), and the average values were used for statistical analysis. BIC (%) was calculated as “bone contact length within threads/overall length of threads.” Bone area (BA, %) was calculated as “area of newly formed bone between threads/overall area between threads.”

### 2.10. Statistical Analysis

Statistical analysis was performed using SPSS version 25 (IBM Corp., Armonk, NY, USA). Normality of all the data were evaluated using the Shapiro–Wilk test. The data were not normally distributed; thus, the Kruskal–Wallis test was used to determine whether the mean values of BIC and BA differed significantly among groups at each time point. This was followed by the Mann–Whitney U test with Bonferroni correction for multiple comparisons. Next, a two-way analysis of variance followed by post hoc Tukey’s test was used to determine whether the mean values of BIC and BA differed significantly between the 2- and 4-week time points in each group. Subsequently, the Mann–Whitney U test was used for multiple comparisons. The level of statistical significance was set at α = 0.05. GraphPad Prism 7.0 software (GraphPad Software Inc., La Jolla, CA, USA) was used to visualize the data.

### 2.11. Ethical Considerations

All procedures, including animal selection, care, preparation, general anesthesia, and surgical steps, were approved by the Institutional Animal Care and Use Committee (Yonsei Medical Center, Seoul, Korea; Approval No. 2019-0157). The housing protocol suggested by the Association for Assessment and Accreditation of Laboratory Animal Care International guidelines was followed. In addition, the study design complied with the ARRIVE guidelines.

## 3. Results

### 3.1. FTIR Spectra Analysis of Titanium Discs

**Figure 5:**
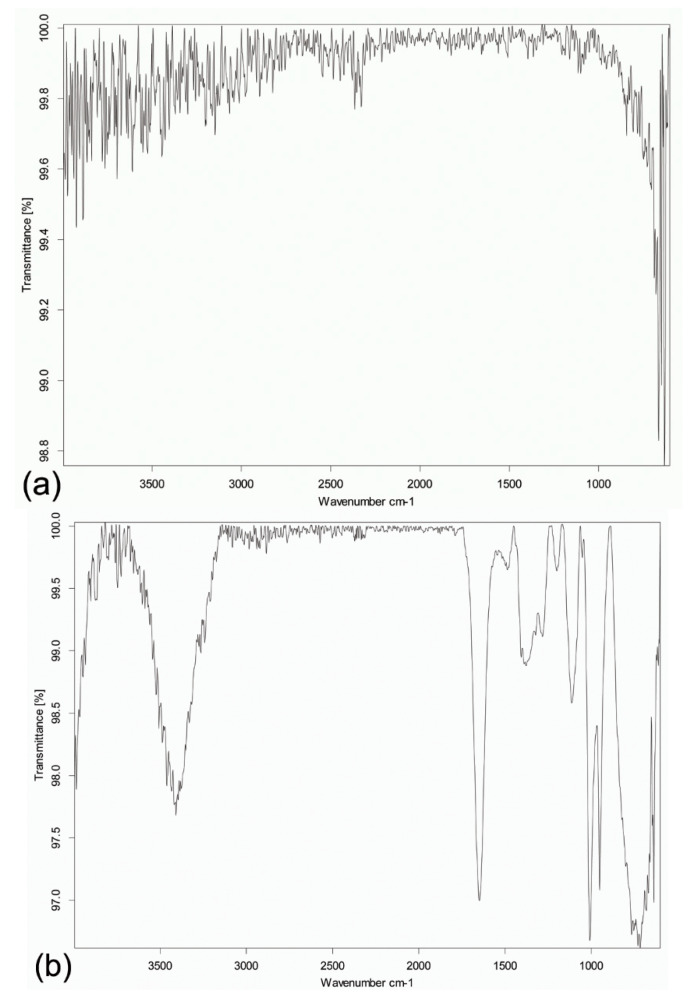
Representative Fourier-transformed infrared spectra of the titanium discs. (**a**) Spectrum of the sandblasted, large-grit, acid-etched surface-treated titanium disc. (**b**) Spectrum of the same kind of disc but further immersed in simvastatin solution. Note the distinct bands of the functional groups OH (3600–3200 cm^−1^) and C=O (1800–1600 cm^−1^), which are present on simvastatin. The characteristic fingerprint region of simvastatin occurring at <1500 cm^−1^ further confirms alteration of the surface of the titanium disc upon simvastatin treatment.

Figure 5 shows the FTIR spectra of the titanium discs. The spectrum of the disc immersed in simvastatin solution resembled that of simvastatin reported in the literature [63,64]. The prominent bands of the OH stretching vibrations (3600–3200 cm^−1^) and the carbonyl C=O stretching vibration (1800–1600 cm^−1^) were observed. The fingerprint region occurring at <1500 cm^−1^ indicated the presence of simvastatin adsorbed onto the titanium surface. The spectral analysis confirmed the alteration of the surface of the titanium disc upon simvastatin treatment from a chemical perspective.

### 3.2. Clinical Assessment of Experimental Animals

During the postsurgical period, all rabbits recovered uneventfully. No signs of the complications stated in the exclusion criteria were observed. After sacrifice, a normal periosteum was found in all specimens, and no clinical signs of adverse tissue reactions were observed at a macroscopic level. All implants were still in situ and available for histological analysis; therefore, all samples were included in subsequent analyses.

### 3.3. Histological Examination and Quantitative Histomorphometry

As both the nongrafted and grafted sides of each implant were examined, 96 sites were analyzed in total. The mean value of the interclass correlation coefficient was larger than 0.8, implying a strong level of agreement between the examiners. The average values of the measurements made by the two examiners were used.

**Figure 6:**
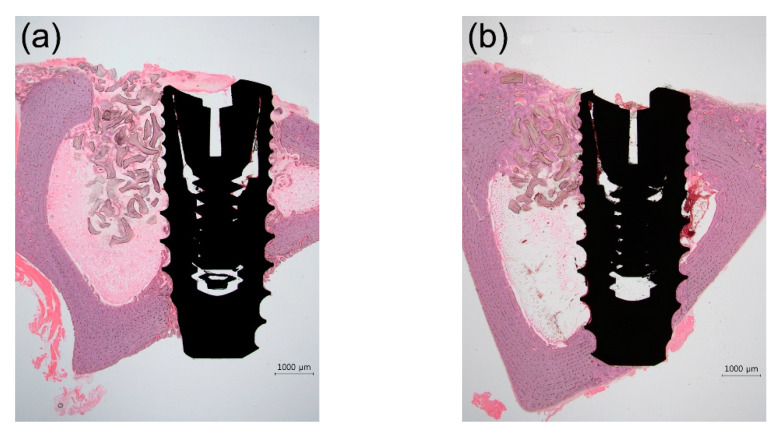
Representative photomicrographs (12.5× magnification) of hematoxylin- and eosin-stained histological sections of rabbit tibias at the implanted regions. (**a**) 2-week group; (**b**) 4-week group. Scale bars: 1000 μm.

**Figure 7:**
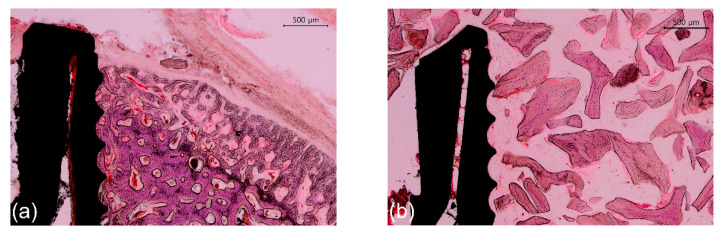

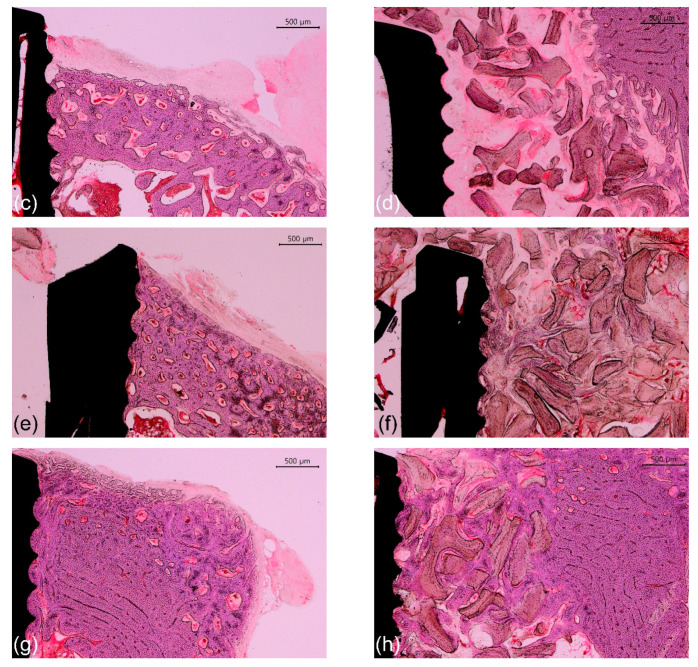
Representative photomicrographs (40× magnification) of hematoxylin and eosin-stained histological sections of rabbit tibias at the implanted regions. (**a**) Group C-2, nongrafted side; (**b**) Group C-2, grafted side; (**c**) Group SU-2, nongrafted side; (**d**) Group SU-2, grafted side; (**e**) Group C-4, nongrafted side; (**f**) Group C-4, grafted side; (**g**) Group SU-4, nongrafted side; and (**h**) Group SU-4, grafted side. Note the sparse amount of new bone on the grafted side in 2-week groups. Scale bars: 500 μm.

**Figure 8:**
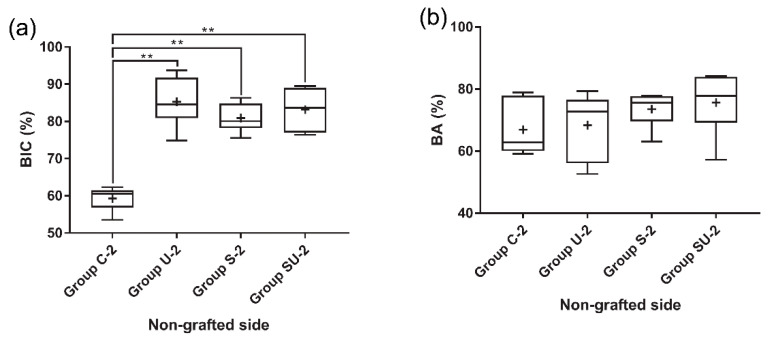

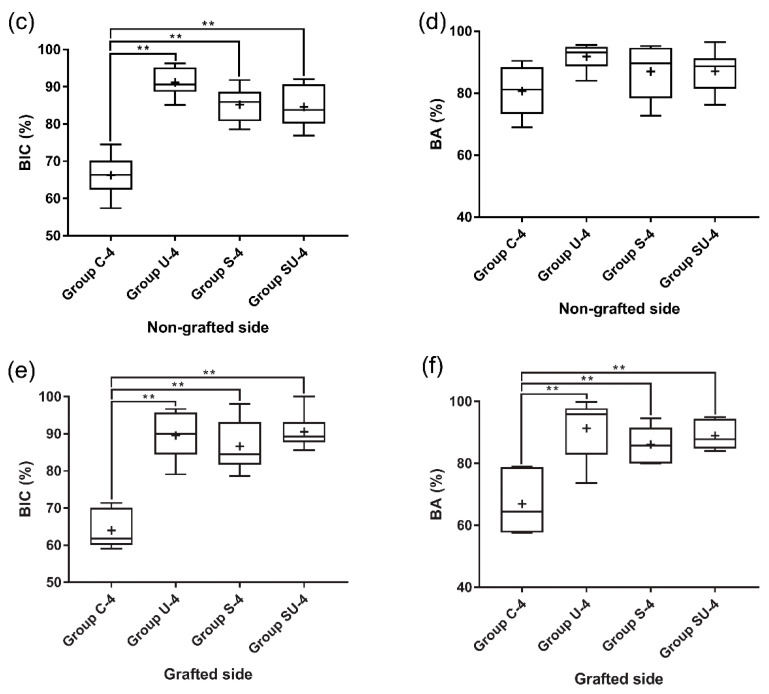
Histomorphometric analyses of the experimental groups. The grafted sides of 2-week groups were not analyzed due to a sparse amount of new bone in the vicinity of the implants. (**a**) BIC at the nongrafted side of the 2-week group; (**b**) BA at the nongrafted side of the 2-week group; (**c**) BIC at the nongrafted side of the 4-week group; (**d**) BA at the nongrafted side of the 4-week group; (**e**) BIC at the grafted side of the 4-week group; and (**f**) BA at the grafted side of the 4-week group. Asterisks indicate statistically significant differences among the groups (** *p* < 0.01). The lines inside the boxes indicate median values, and the cross signs inside the boxes indicate mean values. The borders of the boxes indicate the 25th and 75th percentiles. The whiskers indicate the minimum and maximum values. BIC, bone-to-implant contact; BA, bone area.

**Figure 9:**
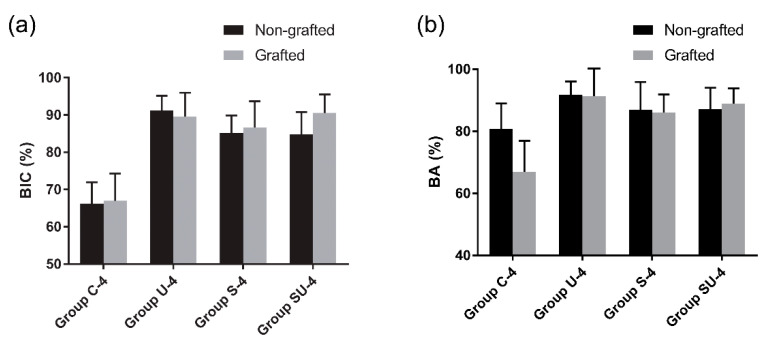
Comparisons between the nongrafted sides and the grafted sides in the 4-week group, as evaluated by histomorphometry. (**a**) BIC; (**b**) BA. Data are expressed as mean ± standard deviations. Error bars show the standard deviations. Note that there is no significant difference between the nongrafted sides and the grafted sides in terms of BIC and BA (*p* > 0.05), regardless of the treatment methods used. BIC, bone-to-implant contact; BA, bone area.

**Figure 10:**
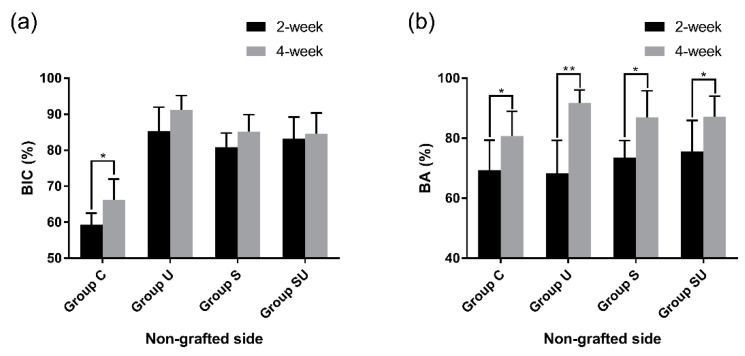
Comparisons between the 2- and the 4-week groups on the nongrafted sides, as assessed by histomorphometry. (**a**) BIC; (**b**) BA. Data are expressed as mean ± standard deviations. Asterisks indicate statistically significant differences among the groups (* *p* < 0.05 and ** *p* < 0.01). Error bars show the standard deviations. BIC, bone-to-implant contact; BA, bone area.

Representative photomicrographs are shown in Figure 6 and Figure 7; the results of the histomorphometric analysis are illustrated in Figure 8, Figure 9 and Figure 10. Apical migration of the epithelium and connective tissue was not observed in any of the samples. In the 2-week group, on the sides with bone grafting, a sparse amount of new bone in the vicinity of the implants was observed in the majority of the specimens, regardless of the method of surface modification used (Figure 7b,d). Therefore, the examiners were unable to perform histomorphometric measurements.

#### 3.3.1. The 2-Week Group

In the nongrafted sides, bone neoformation was found at the bone/implant interface in cortical bone (Figure 7a,c). The mean BIC values of group U-2, S-2, and SU-2 were 85.2%, 80.9%, and 83.2% respectively. These values were significantly higher (approximately 20%) than the BIC value (59.3%) of group C-2 (*p* < 0.05) (Figure 8a). There was no statistically significant difference in the BIC values among the experimental groups (*p* > 0.05). The BA values did not differ significantly among the four groups (*p* > 0.05) (Figure 8b).

#### 3.3.2. The 4-Week Group

In the sides contacting the innate bone, the amount of newly formed bone in direct contact with the implant surface was more prominent in comparison with that in the 2-week group (Figure 7e,g). The mean BIC values of group U-4, S-4, and SU-4 were 91.2%, 85.2%, and 84.6%, respectively. Similar to the 2-week group, the BIC values of group U-4, S-4, and SU-4 were approximately 20% higher than the BIC value (66.2%) of group C-4 (*p* < 0.05) (Figure 8c). There was no statistically significant difference in the BIC values among the experimental groups (*p* > 0.05). The BA values did not differ significantly among the four groups (*p* > 0.05) (Figure 8d). The overall numerical tendency was analogous to that of the 2-week group.

On the sides with bone grafts, newly formed bone was observed at the bone/implant interface and around the biomaterial (Figure 7f,h). The bone graft materials were not degraded during this period and were discernible from the newly formed bone. Both the BIC and BA values of group U-4, S-4, and SU-4 were significantly larger (about 20%) than those of group C-4 (*p* < 0.05) (Figure 8e,f). There was no significant difference in either parameter among group U-4, S-4, and SU-4 (*p* > 0.05). When the nongrafted and grafted sides in the 4-week group were compared, there was no significant difference between them in terms of BIC and BA (*p* > 0.05), regardless of the surface treatment methods used (Figure 9).

#### 3.3.3. Comparison of the 2-Week and 4-Week Groups

In the nongrafted sides, group C exhibited a significant increase in both BIC (about 10% higher) and BA (about 20% higher) (*p* < 0.05) (Figure 10). In contrast, the group U, S, and SU displayed a significant increase in BA (about 20% higher) only (*p* < 0.05).

## 4. Discussion

The null hypothesis of the present study was rejected because UV treatment and/or simvastatin immersion of implants resulted in considerable bone formation in vivo at the bone-implant interface, both in surfaces contacting the pristine bone and in the neighboring gap defects filled with xenogenic bone substitutes. To the best of our knowledge, no previous study has integrated UV-treated and simvastatin-saturated implants along with bone-grafting procedures in peri-implant defects or compared the effects of UV, simvastatin, and the combination of both.

Healing periods of 2 and 4 weeks were adopted based on previous studies [27,52,65]. To measure the efficacy of UV and/or simvastatin to facilitate osseointegration, the time window was set earlier than the time required for a titanium implant to be osseointegrated in a rabbit long bone under normal conditions [66,67]. Our results showed that, in the nongrafted sides, a higher degree of osseointegration in the experimental groups (group U, S, and SU) was observed at 2 and 4 weeks postimplantation in rabbits, as demonstrated by the BIC values. Therefore, modification of the implant surface by UV pretreatment or simvastatin immersion seems to enhance bone healing and shorten the healing time for osseointegration.

In the 2-week group, on the grafted sides, a scarce amount of new immature bone was formed between the implant threads to perform histometric analysis, implying that 2 weeks of healing was not long enough. This is in agreement with a study by Araújo et al. [68], in which a delay in bone repair was reported for extraction sockets filled with bone substitutes. It could be inferred that, on the grafted side, the implant surface is distant from the osteogenic sources; that is, the pristine bone, which results in a relative lack of new bone formation during such a short period. Nevertheless, in the 4-week group, both BIC and BA could be measured, and the experimental groups presented higher values. This shows that UV illumination or simvastatin immersion promoted osteogenic potential around the implants in the presence of bone graft materials, even under conditions in which only a few cells and limited blood supply exist.

In both 2- and 4-week groups, in the nongrafted sides, there was no significant difference in the BA values between groups, whereas the BIC values differed significantly between group C and the experimental groups (group U, S, and SU). Moreover, in each group using a defined treatment option, there were statistically significant differences between the 2- and 4-week time points on the nongrafted sides: both BIC and BA in the control group; only BA in the experimental groups. Davies [69] described two different osteogenesis phenomena around dental implants: distance osteogenesis and contact osteogenesis. Distance osteogenesis occurs on the surface of an existing peri-implant bone through appositional growth, progressing toward the implant surface. Contact osteogenesis is de novo bone formation that occurs on the bioactive surface of the implant, and the orientation of bone growth is from implant to bone. On the nongrafted sides, distance osteogenesis from the nearby native bone would have greatly influenced new bone formation. Consequently, we presume that there was no significant difference in the BA values between the experimental and the control groups on those sides after 2 and 4 weeks postoperatively, respectively. The finding that the BIC values had already reached a plateau at 2 weeks in the experimental groups may be due to active contact osteogenesis resulting from the surface modification. This suggests that the effects of UV or simvastatin treatment of implants mainly occurred before 2 weeks in these sites.

In our study, simvastatin was selected because of its widely proven effects in many studies. To date, numerous studies have shown that statins strongly modulate bone anabolism in multiple ways [40,41,42,43]. However, unlike bisphosphonates, statins have a lower bone affinity, and much higher clinical doses than the lipid-lowering therapy are required to exert an influence on bone healing, resulting in greater chances of systemic off-target effects of statins [43]. Through local delivery, the bioavailability of statins is increased, and the dose required for osseous healing is reduced. Consequently, the possibility of developing complications from systemic simvastatin use is lowered [27,43]. The local delivery method could increase the cost effectiveness by reducing the amount of simvastatin administered, further adding to its advantages. By immersing implants in simvastatin solution, we can simply and safely apply a low concentration of simvastatin and acquire the desired outcomes in the peri-implant area.

According to a recent systematic review, implants that were treated with simvastatin solution all offered significantly better results in the parameters related to osteogenesis in rat studies [47]. There are several ways of local delivery suggested to date: impregnation, repeated local injection, and the use of carriers. In the case of impregnation, the drug is mostly available during the first hours of application. Although local injection may be more reliable, repeated injections may be traumatic, hindering the healing process and carrying the risk of contamination. Conversely, using carriers allows for the release of drugs at a controlled rate and concentration. For example, the local application of simvastatin in poly-γ-glutamic acid gel has shown anabolic effects on the bone around titanium implants in rats [70]. However, in most cases, drug carriers are costly and complicated to produce. Drug vehicles are still under development and have not been extensively used in clinical settings. We chose impregnation because it is simple, cost-effective, readily applicable, and does not require additional patient visits in clinical settings.

We set the concentration of simvastatin to 0.5 mM, which is higher than that used by other studies [51,71,72,73,74]. The reason for this choice was that simvastatin was directly applied to the implant surface, resulting in a relatively high concentration, therefore achieving an effective concentration for local delivery. The implants were homogenously wetted with simvastatin solution for 24 h before implantation. The simvastatin-coated implants would have produced a burst release of simvastatin, generating a high concentration of the drug at the initial phase of osseous healing; however, simvastatin is only available for the first few hours. Future studies should focus on optimizing the concentration and associated local delivery method of simvastatin in clinical usage, which would form high-quality bone more rapidly and minimize the risk of undesired host/tissue reactions.

Higher BIC and BA values on the grafted sides were found in group S-4 compared to group C-4. This suggests that statins may be incorporated into bone augmentation techniques during implant surgery to achieve adequate bone quality and volume in a short time. To the authors’ knowledge, this was the first implant study to use simvastatin in conjunction with xenogenic bone graft material. Implants coated with simvastatin add osteoinductivity to xenogenic bone substitutes, which are known to possess only osteoconductivity only. The combined effect of simvastatin and xenogenic bone material resulted in superior osseointegration. This result is supported by a rabbit study by Wong and Rabie [24], who concluded that statin could be used alone in small defects or together with other bone grafting materials to exert their osteoinductive effect in larger defects.

Moreover, the SLA surface-treated titanium implants were used in the present study, in line with similar studies that used simvastatin solution to facilitate osseointegration. In rat tibias, Yang et al. [75] placed implants that were roughened by large-grit blasting and acid etching; these were subsequently immersed in simvastatin solutions for drug adsorption onto implant surfaces. They observed that it improved osseointegration in the treated groups. Fang et al. [76] observed that large-grit blasted and acid-etched implants showed improved osseointegration in rat tibias when implant surfaces were coated with simvastatin. In agreement with those studies, we observed that simvastatin-soaked SLA surface implants showed an improvement in osseous repair. Because the SLA surface has more wettability and surface energy than the machined surface, it may have facilitated increased adsorption of simvastatin molecules onto surfaces, consequently leading to better osseointegration [19].

To date, several different mechanisms of UV photofunctionalization have been proposed. It was suggested that UV irradiation removes hydrocarbon contaminants from the titanium surface, which contributes to protein adsorption and cell attachment enabling more rapid osseointegration [20,21]. Additionally, UV-initiated photocatalytic activity alters the surface of grade IV titanium to produce lower reactive oxygen species levels than untreated titanium, providing a favorable environment for cellular adsorption and proliferation [28]. Moreover, it should be noted that UV irradiation is a well-known sterilization technique. The decreased likelihood of infection may have promoted the healing process. An in vitro study confirmed that UV treatment of titanium prior to installation decreases the capability of human oral bacteria to form colonies in the presence of salivary and blood components [77]. The multibeneficial actions of UV treatment would have contributed to the significantly accelerated bone healing in the UV-treated groups in our study.

Ueno et al. [22] described the use of UV-treated implants in a gap defect. They placed titanium rods that were treated with UV into a rat femur without being in contact with the innate cortical bone. They confirmed that the UV-treated implants in the gap healing condition resulted in bone–titanium integration with a strength equal to that of untreated implants in the contact healing condition. Accordingly, our study indicated that UV treatment of titanium increased the osteogenic potential effectively, even in an environment that lacks contact and support from the natural bone tissue. It could be stated that UV treatment was compatible with the xenogenic bone graft material used in our study.

In our study, simvastatin immersion followed by UV illumination did not show a synergistic effect. Group SU did not yield statistically significantly better results than group U or S. We assume that UV irradiation may have decomposed simvastatin. Simvastatin is an organic compound composed of carbon skeletons, and it is probable that UV has broken down carbon skeletons by photon-induced radical reactions, the main mechanism by which UV cleanses the titanium surface. Our result is in disagreement with that of Kim et al. [27], who observed a synergistic effect of UV treatment and alendronate soaking in implants placed in rabbit tibias. If the implants were first UV-treated and then immersed in simvastatin solution, we speculated that the effect of UV might fade away due to the aging of titanium. Our study implies that UV treatment is probably not compatible with organic compound coatings on the implant surface unless there is a special method to eliminate hydrocarbon contaminants on the titanium surface selectively.

This study is liable to a few limitations. Due to the small number of samples per group and the usage of nonparametric statistical analyses, the statistical power may have been affected, and therefore the results should be interpreted with caution. Although a single experienced operator placed implants under similar circumstances, the surgical technique could have influenced the outcomes. Each group of implants was placed in the predetermined tibial location and side. This pre-allocated anatomical position may have influenced the result due to subtle differences in bone tissue and muscle movement. The differences among the experimental groups regarding bone-regeneration advancement may have been appreciated more thoroughly by applying other staining techniques, such as Alizarin Red and Von Kossa stains. Identifying bone-related molecular markers, including osteopontin and osteocalcin, would provide further data on the extent of the osseointegration process. Moreover, the absence of occlusal forces, the histological differences between the rabbit tibia bone and human jawbones, and the low chance of infection may introduce bias when interpreting the results [47].

## 5. Conclusions

Within the limitations of this study, for a peri-implant bone defect in the rabbit tibia, histomorphometric analysis supported that UV irradiation or immersion of the implant in simvastatin solution has the potential to promote the rate of bone regeneration around titanium implants, as characterized by significantly higher BIC values. However, no synergistic effect of the two treatments was observed. Based on our findings, we suggest that UV photofunctionalization or simvastatin immersion may be helpful in accelerating implant loading periods and increasing implant stability in a shorter time when placing implants in freshly extracted sockets with osteoconductive grafts. Both treatments may function as supplements to clinicians encountering the challenges of immediate implantation and might lead to novel strategies for early implant restorations.

## Data Availability

The data presented in this study are available on request from the corresponding author.
