# Correction: Jun et al. Improvement of Osseointegration by Ultraviolet and/or Simvastatin Treatment on Titanium Implants with or without Bone Graft Materials. *Materials* 2021, *14*, 3707

**DOI:** 10.3390/ma15041293

**Published:** 2022-02-10

**Authors:** Ji Hoon Jun, Kyung Chul Oh, Kyu-Hyung Park, Narae Jung, Jiayi Li, Hong Seok Moon

**Affiliations:** 1 Department of Prosthodontics, Yonsei University College of Dentistry, Seoul 03722, Korea; jhjun0103@yonsei.ac.kr (J.H.J.); kyungabc@yuhs.ac (K.C.O.); ljyzoeli24@gmail.com (J.L.); 2 Aeromedical Squadron, Republic of Korea Air Force 8th Fighter Wing, Wonju 26304, Korea; 3 Oral Science Research Center, BK21 Plus Project, Yonsei University College of Dentistry, Seoul 03722, Korea; khyungpark@gmail.com (K.-H.P.); jnrgood1217@yuhs.ac (N.J.)

## Additional Affiliation

In the published publication [1], there was an error regarding the affiliations for Ji Hoon Jun. In addition to affiliation 1, “Aeromedical Squadron, Republic of Korea Air Force 8th Fighter Wing, Wonju 26304, Korea” should be added. The corrected affiliation appears above.

The authors apologize for any inconvenience caused and state that the scientific conclusions are unaffected. The original publication has also been updated.
